# Impact of 8-week linoleic acid intake in soy oil on Lp-PLA_2_ activity in healthy adults

**DOI:** 10.1186/s12986-017-0186-2

**Published:** 2017-05-08

**Authors:** Minkyung Kim, Minjoo Kim, Ayoung Lee, Hye Jin Yoo, Jung Soo Her, Sun Ha Jee, Jong Ho Lee

**Affiliations:** 1 Research Center for Silver Science, Institute of Symbiotic Life-TECH, Yonsei University, Seoul, 03722 Korea; 2 National Leading Research Laboratory of Clinical Nutrigenetics/Nutrigenomics, Department of Food and Nutrition, College of Human Ecology, Yonsei University, Seoul, 03722 Korea; 3 Department of Food and Nutrition, Brain Korea 21 PLUS Project, College of Human Ecology, Yonsei University, Seoul, 03722 Korea; 4 Institute for Health Promotion, Graduate School of Public Health, Yonsei University, Seoul, 03722 Korea

**Keywords:** Linoleic acid, Soybean oil, Lp-PLA_2_, Cardiovascular disease, CEPI-CT

## Abstract

**Background:**

No intervention follow-up study has examined the association between plasma n-6 polyunsaturated fatty acids (PUFAs) and lipoprotein-associated phospholipase A_2_ (Lp-PLA_2_), which is a risk factor for cardiovascular disease (CVD). We aimed to determine whether the administration of linoleic acid (LA, 18:2n-6) in soy oil affected Lp-PLA_2_ activity in healthy adults.

**Methods:**

Self-reported healthy participants (*n* = 150) were randomly assigned to three groups: a low LA group, in which 10 mL soy oil was replaced with one apple; a medium LA group, in which the typical food intake was maintained; and a high LA group, in which 1/3 cup of cooked refined rice was replaced with 9.9 g of soy oil capsules daily. Plasma fatty acids and Lp-PLA_2_ activity were measured along with other CVD risk factors.

**Results:**

After 8 weeks of treatment, plasma LA levels decreased in the low LA group and increased in the high LA group. The high LA group showed greater increases in apolipoprotein B (apoB) and oxidized low-density lipoprotein (ox-LDL) than those in the low LA group. Plasma LA levels and Lp-PLA_2_ activities demonstrated greater increases in the high LA group than those in the medium and low LA groups. Changes in plasma LA positively and independently correlated with changes in Lp-PLA_2_ activity, which was negatively correlated with changes in collagen-epinephrine closure time (CEPI-CT).

**Conclusions:**

An increase in plasma LA following intake of soy oil was independently associated with Lp-PLA_2_ activity, which was also related to apoB, ox-LDL and CEPI-CT.

**Trial registration:**

ClinicalTrail.gov Identifier: NCT02753907, registered 25 April 2016 (retrospectively registered).

**Electronic supplementary material:**

The online version of this article (doi:10.1186/s12986-017-0186-2) contains supplementary material, which is available to authorized users.

## Background

Lipoprotein-associated phospholipase A_2_ (Lp-PLA_2_) is a macrophage-derived enzyme that contributes to oxidative stress, vascular inflammation, and endothelial activation [1, 2]. Elevated levels of Lp-PLA_2_ have been associated with unfavorable health outcomes, including an increased risk for myocardial infarction and cardiovascular disease (CVD)-related deaths in adults [3]. Indeed, our previous study indicated that elevated Lp-PLA_2_ activity was associated with prehypertension [4], which reflects an early increased risk of CVD [5]. Furthermore, several studies on darapladib, a drug that inhibits Lp-PLA_2_ activity, showed its beneficial effect on CVD in accordance with decreases in Lp-PLA_2_ activity; Mohler et al. [6] reported that darapladib dose-dependently decreased interleukin (IL)-6 in patients with stable coronary disease; Serruys et al. [7] indicated that its administration to patients with angiographically proven coronary disease inhibited the increase in necrotic core volume (coronary atheroma volume) compared to that of individuals who were not given the treatment. However, the role of Lp-PLA_2_ in CVD is controversial; Blake et al. [8] showed that Lp-PLA_2_ activity did not predict CVD events in women after adjusting for traditional risk factors; Rosenson and Stafforini [9] reported that it was unclear whether a mechanism regarding limit necrotic core expansion by darapladib is a consequence of Lp-PLA_2_ activity inhibition. Moreover, the researchers demonstrated that Lp-PLA_2_ activity did not predict future risk in individuals who had adequately managed cholesterol levels in several intervention studies [9]. Thus, the role of Lp-PLA_2_ as a risk factor of CVD is unclear.

Omega-3 polyunsaturated fatty acid (n-3 PUFA) supplementation reduces markers of inflammation and endothelial dysfunction, which are risk factors of CVD, including atherosclerosis [10]. However, consumption of large amounts of omega-6 (n-6) PUFAs had proinflammatory, prothrombotic, and proaggregatory effects and promoted the formation of thrombus and atheromas [11]. Thus, we hypothesized that there is a link between intake of n-6 PUFAs and Lp-PLA_2_ activity for the increased risk of CVD. A number of follow-up intervention trials have been performed to examine Lp-PLA_2_ activity in the context of n-3 fatty acids. However, no intervention study has examined the association between changes in plasma n-6 fatty acids and Lp-PLA_2_ activity, which is recognized as an independent risk factor for CVD [2]. Recently, Steffen et al. [12] reported that n-6 fatty acids were independently associated with Lp-PLA_2_ in their cross-sectional, multi-ethnic study of atherosclerosis. Therefore, the present follow-up intervention study aimed to determine whether 8 weeks of linoleic acid (LA, C18:2n-6) representing n-6 PUFA administration in the form of soy oil would alter Lp-PLA_2_ activity, as well as other CVD risk factors, in healthy adults.

## Methods

### Subjects

Subjects were recruited via a poster advertisement in Seoul, Korea, between June and September of 2015. Subjects who agreed to participate were screened for their health status. Blood samples were obtained to evaluate their clinical and biochemical parameters, and self-reported history of diagnosis and treatment of any disease, as well as intake of medication or supplements, was also examined. After the screening, 150 subjects aged 30–65 years, who were non-diabetic (fasting blood glucose < 126 mg/dL) and non-obese [a body mass index (BMI) between 18.5 kg/m^2^ and 30 kg/m^2^], had no history of any disease, and were not using any medication or supplements, were finally enrolled in this study. Exclusion criteria included dyslipidemia, diabetes mellitus, hypertension, liver disease, renal disease, chronic disease of the gastrointestinal tract, cerebrovascular disease, pancreatitis, cancer, intake of any medication or supplements, and women who were pregnant or lactating. Drug or alcohol abuse (alcohol consumption >280 g/week for men, >140 g/week for women) also excluded participation in the intervention. Written informed consent was obtained from all participants, and the Institutional Review Board of Yonsei University approved the study protocol, which complied with the Helsinki Declaration.

### Study design and intervention

An 8-weeks, randomized, placebo-controlled study was conducted with 150 healthy adults. The participants were divided into three groups: a low LA group composed of 50 individuals, in which 10 mL soy oil was replaced with one apple; a medium LA group (control group) composed of 50 individuals, in which the typical food intake was maintained; and a high LA group composed of 50 individuals, in which 1/3 cup of cooked refined rice was replaced with 9.9 g (9 1.1 g capsules, 3 per meal) of soy oil daily as a supplement (ClinicalTrials.gov: NCT02753907; http://www.clinicaltrials.gov) [13]. The soy oil capsules were provided by Misuba RTech Co., Ltd. (Asan, Korea). The soy oil used in this study contained 54.2% LA, 21.6% oleic acid, 10.7% palmitic acid, 8.1% α-linolenic acid, and 4.1 mg of α-tocopherol/100 g [14, 15]. The subjects kept food diaries 3 days before the baseline visit and for 3 days during weeks 4 (middle of intervention) and 8 (end of intervention). Individual sessions lasted 1 h and included supportive counseling and dietary instructions. Group sessions were held separately for participants in the low LA and high LA groups. The participants received the same dietary behavioral curriculum, which included identical information on dietary fiber intake, education on the food pyramid and portion control. The daily low LA and high LA replacement items (apple or capsules) were provided to participants in each group for the duration of the intervention. Participants were counseled to maintain their baseline levels of physical activity, which was assessed using validated measures at each visit. Compliance was assessed via the dietary intake record along with the amount of unconsumed oil capsules or apples returned at the middle and final visits.

### Daily energy intake and physical activity measurements

Details regarding daily energy intake and physical activity have been previously published [16]. Briefly, a standardized 3-days dietary record (2 weekdays and 1 weekend day) was obtained from each participant. This record was completed at home after the participants received detailed explanations from a dietitian. This measurement was performed during weeks 0, 4, and 8. A computerized version of the Korean Nutrition File (Can-Pro 3.0; The Korean Nutrition Society, Seoul, Korea) was used to determine the macronutrient content of the foods consumed by the participants and their total daily energy intake. In addition, the participants completed a semi-quantitative food frequency questionnaire and a 24-h recall with the assistance of a dietitian on weeks 0, 4, and 8 to confirm the accuracy of the dietary record. A standardized 3-days physical activity record was also completed at home on the same days that the dietary record was completed, and total energy expenditure was calculated.

### Anthropometric parameters and blood pressure

Detailed information on these parameters is provided in our previous paper [16]. Briefly, body weight, height and waist circumference were measured at screening, baseline and the 8-weeks follow-up visit. BMI was calculated in units of kilograms per square meter (kg/m^2^). During each testing session, systolic and diastolic blood pressure values were measured in a supine position after a resting period.

### Biochemical analyses and plasma Lp-PLA_2_ activity and oxidized LDL

Detailed information has been previously published [16]. Briefly, blood samples were collected following an overnight fast of at least 12 h, and the levels of fasting triglycerides; total-, high-density lipoprotein (HDL)-, and low-density lipoprotein (LDL)-cholesterol; apolipoprotein (apo) A-I and B; glucose; insulin; and high-sensitivity C-reactive protein (hs-CRP) were measured. The activity of Lp-PLA_2_, also known as platelet-activating factor acetylhydrolase (PAF-AH), was measured using a PAF-AH activity assay kit (Biovision, Milpitas, CA). The resulting change in absorbance was immediately read at 412 nm for 30 min at room temperature using a VERSAmax microplate reader in kinetic mode (Molecular Devices, Sunnyvale, CA). Lp-PLA_2_ activity was expressed in nmol of PAF hydrolyzed per min per mL of serum. Plasma oxidized LDL (ox-LDL) was measured using an enzyme immunoassay (Mercodia AB, Uppsala, Sweden). The resulting color reaction was monitored at 450 nm with a Wallac 1420 Victor^2^ multilabel counter (PerkinElmer Life Sciences, Boston, MA).

### Hemostasis tests

The details of the hemostasis tests have been previously published [16]. Blood samples used for the platelet function analyzer-100 (PFA-100; Siemens, Marburg, Germany) assay were collected in 2.7 mL tubes containing 3.2% sodium citrate and analyzed independently. Collagen-epinephrine closure time (CEPI-CT) was analyzed using a PFA-100 system according to the manufacturer’s instructions. Prothrombin time (PT) and activated partial thromboplastin time (aPTT) were measured using a Sysmex CA-1500 coagulation analyzer (Sysmex, Nagano, Japan). The fibrinogen concentration was determined via a light scattering method with the Sysmex CA-1500 coagulation analyzer.

### Fatty acid concentrations using gas chromatography mass spectrometry (GC-MS)

The details of GC-MS have been previously published [16]. Briefly, all analyses were performed on an Agilent Technologies 7890 N gas chromatograph coupled to an Agilent Technologies 5977A quadrupole mass selective spectrometer with a triple-axis detector (Agilent, Palo Alto, CA) in the electron ionization mode (70 eV) and full scan monitoring mode (m/z 50–800). Derivatized samples were separated on a VF-WAX column (Agilent Technologies, Middelburg, Netherlands) with helium as the carrier gas and a temperature ramp from 50 °C to 230 °C. Metabolites in the samples were identified by comparing their relative retention times and mass spectra with those of authentic reference standards. The relative metabolite levels were calculated by comparing their peak areas to that of the internal standard compound.

### Statistical analysis

Statistical analysis was performed using SPSS version 21.0 (IBM/SPSS, Chicago, IL). Logarithmic transformation was performed on skewed variables. For descriptive purposes, the mean values are presented using untransformed values. The results are expressed as the mean ± standard error. A two-tailed *P*-value <0.05 was considered statistically significant. We compared the parameters at baseline and at the 8-weeks follow-up visit, and the net change (difference from baseline) among the low, medium, and high LA groups was assessed using one-way analysis of variance (ANOVA) with a Bonferroni post hoc test. Paired *t*-tests were performed to compare the effects of the intervention within each group. A stepwise multiple regression analysis was performed to identify the major variables influencing Lp-PLA_2_ activity. Pearson’s correlation coefficient was used to examine relationships between variables. A heat map was generated to visualize correlations among variables.

## Results

Among the enrolled subjects (*n* = 150), 3 dropped out for personal reasons, and 147 subjects completed the study. Among the dropouts, all 3 were in the control (medium LA) group and maintained their usual dietary intake. Thus, the compliance rate based on returned capsules and apples was high (98.5%), and no adverse reactions involving supplementation with soy oil were observed. The soy oil used in this study contained 54.2% LA, 21.6% oleic acid 10.7% palmitic acid, 8.1% α-linolenic acid and 4.1 mg of α-tocopherol/100 g [14, 15]. There were no reports of adverse events from study participants in all three groups during the study period.

### Clinical characteristics

**Fig. 1:**
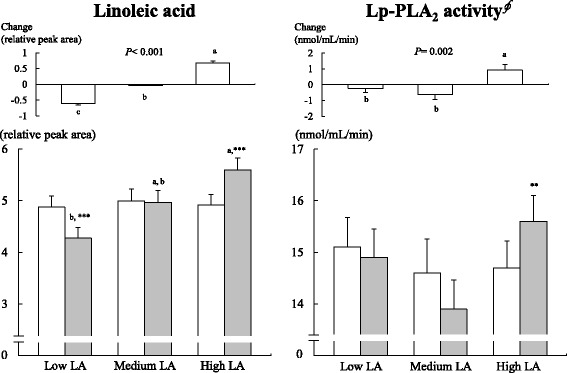
Plasma levels of LA and Lp-PLA_2_ activity at baseline (□) and 8 weeks of follow-up (■) according to LA intake. Mean ± SE. Data included 50 (low LA), 47 (medium LA) and 50 (high LA) participants. ^*§*^Values were tested by logarithmic transformation. *P*-values were derived from ANOVA. All alphabetical *P* < 0.05 values were derived from ANOVA with the Bonferroni post hoc test in follow-up and changed values; no significant changes among each group are indicated with the same letter, while significant changes among each group are indicated with a different letter. ^*^ *P* < 0.05, ^**^ *P* < 0.01, ^***^ *P* < 0.001 indicate comparisons with baseline values in each group performed with a paired *t*-test

No significant differences in the baseline measurements between the 3 dietary groups were found for age, BMI, daily energy intake, % carbohydrate, % protein, and % fat of total energy, blood pressure, lipid profiles, glucose, insulin, hemostatic markers and plasma levels of the four n-6 and three n-3 PUFAs (Table 1 and Fig. 1). After the intervention period, carbohydrates substantially increased in the low LA group as they were given an apple instead of LA supplements, whereas they significantly decreased in the high LA group, who consumed extra LA instead of 1/3 cup of cooked refined rice. The means of the dietary fat intake were broadly met, with changes of −2.3, −0.1, and 3.4% in the respective diets (low LA group, medium LA group and high LA group) relative to the baseline intake. During the intervention, the ratio of PUFAs to saturated fatty acids significantly increased from 1.20 to 1.75 in the high LA group; thus, all dietary characteristics showed the expected changes. There were no significant differences in physical activity levels among the three groups at baseline, 4, and 8 weeks.

**Table 1:** Clinical and biochemical characteristics and plasma levels of fatty acids of each groups according to linoleic acid intake

	Low LA (*n* = 50)		Medium LA (*n* = 47)		High LA (*n* = 50)		*P* ^*a*^	*P* ^*b*^	*P* ^*c*^
	Baseline	Follow-up	Baseline	Follow-up	Baseline	Follow-up			
Age (year)	52.7 ± 0.99		52.0 ± 1.13		52.7 ± 1.13		0.861		
Male/Female n, (%)	9 (18.0)/41 (82.0)		5 (10.6)/42 (89.4)		9 (18.0)/41 (82.0)		0.519		
BMI (kg/m^2^)	23.9 ± 0.41	23.8 ± 0.41	24.4 ± 0.46	24.4 ± 0.46	24.5 ± 0.43	24.5 ± 0.43	0.530	0.481	
Estimated energy intake (kcal/d)^*†*^	2093.3 ± 30.2	2103.4 ± 24.7	2106.0 ± 23.0	2095.9 ± 24.6	2094.8 ± 24.0	2097.4 ± 30.8	0.993	0.978	
Carbohydrate (% of energy)^*†*^	61.7 ± 0.11	64.5 ± 0.32^a,***^	61.4 ± 0.16	61.2 ± 0.23^*b*^	61.8 ± 0.10	58.1 ± 0.40^c,***^	0.080	<0.001	
Protein (% of energy)^*†*^	16.5 ± 0.10	16.2 ± 0.45	16.4 ± 0.15	16.8 ± 0.18	16.5 ± 0.12	16.7 ± 0.14	0.858	0.381	
Fat (% of energy)^*†*^	21.9 ± 0.11	19.6 ± 0.38^c,***^	22.1 ± 0.07	22.0 ± 0.09^b^	22.0 ± 0.10	25.4 ± 0.40^a,***^	0.373	<0.001	
PUFA/SFA^*†*^	1.24 ± 0.02	1.30 ± 0.04^b^	1.23 ± 0.01	1.22 ± 0.01^b^	1.20 ± 0.01	1.75 ± 0.01^a,***^	0.389	<0.001	
n-6/n-3^*†*^	9.79 ± 0.28	9.25 ± 0.36^b^	9.75 ± 0.37	9.66 ± 0.34^a,b^	9.83 ± 0.26	10.7 ± 0.26^a,*^	0.928	0.003	
γ-linolenic acid (C18:3, n-6)^*‡*^	0.194 ± 0.017	0.162 ± 0.011^*^	0.185 ± 0.011	0.182 ± 0.010	0.176 ± 0.016	0.208 ± 0.020^*^	0.692	0.070	
Change	−0.032 ± 0.014^b^		−0.003 ± 0.007^a,b^		0.032 ± 0.015^a^				0.001
α-linolenic acid (C18:3, n-3)^*‡*^	0.139 ± 0.018	0.120 ± 0.011^b^	0.125 ± 0.010	0.129 ± 0.011^b^	0.159 ± 0.017	0.188 ± 0.021^a^	0.319	0.004	
Change	−0.020 ± 0.013^b^		0.004 ± 0.004^a,b^		0.029 ± 0.015^a^				0.018
Dihomo-γ-linolenic acid (C20:3, n-6)^*‡*^	0.146 ± 0.005	0.130 ± 0.007^b,**^	0.154 ± 0.005	0.155 ± 0.005^a^	0.142 ± 0.007	0.157 ± 0.008^a,*^	0.345	0.008	
Change	−0.016 ± 0.005^b^		0.001 ± 0.005^a,b^		0.014 ± 0.005^a^				<0.001
Arachidonic acid (C20:4, n-6)^*‡*^	0.647 ± 0.016	0.632 ± 0.017	0.666 ± 0.019	0.670 ± 0.019	0.654 ± 0.020	0.691 ± 0.019^**^	0.753	0.062	
Change	−0.015 ± 0.015^b^		0.004 ± 0.011^a,b^		0.038 ± 0.013^a^				0.015
Eicosapentaenoic acid (C20:5, n-3)^*‡*^	0.233 ± 0.016	0.236 ± 0.015	0.230 ± 0.018	0.239 ± 0.016	0.211 ± 0.017	0.197 ± 0.011	0.604	0.071	
Docosahexaenoic acid (C22:6, n-3)^*‡*^	0.428 ± 0.025	0.411 ± 0.023	0.446 ± 0.030	0.440 ± 0.028	0.458 ± 0.024	0.455 ± 0.019	0.715	0.404	
Systolic BP (mmHg)	123.1 ± 1.82	120.5 ± 1.76	120.2 ± 1.92	117.8 ± 1.76	122.1 ± 1.78	123.1 ± 1.77	0.543	0.106	
Diastolic BP (mmHg)	79.5 ± 1.42	76.9 ± 1.24^*^	76.8 ± 1.34	74.5 ± 1.23^*^	77.4 ± 1.22	77.3 ± 1.26	0.333	0.243	
Prothrombin time (sec)	10.5 ± 0.07	10.6 ± 0.08	10.7 ± 0.08	10.7 ± 0.08	10.6 ± 0.08	10.6 ± 0.07	0.552	0.730	
Activated partial thromboplastin time (sec)	26.3 ± 0.37	26.9 ± 0.42^*^	27.3 ± 0.35	27.4 ± 0.40	26.4 ± 0.33	26.5 ± 0.35	0.103	0.288	
Fibrinogen (mg/dL)	260.7 ± 5.12	272.1 ± 6.54^*^	269.0 ± 6.51	270.9 ± 6.33	267.4 ± 5.27	263.0 ± 4.63	0.541	0.491	
CEPI-CT (sec)	142.4 ± 7.37	144.4 ± 8.20	141.2 ± 5.50	141.4 ± 7.88	151.3 ± 8.41	144.2 ± 7.26	0.561	0.694	
Triglyceride (mg/dL)^*∮*^	132.3 ± 10.2	117.4 ± 8.38	123.1 ± 8.57	122.1 ± 9.71	136.8 ± 12.7	169.6 ± 28.3	0.938	0.137	
Total-cholesterol (mg/dL)^*∮*^	228.3 ± 3.44	210.1 ± 4.40^b,***^	231.2 ± 3.91	227.5 ± 3.90^a^	222.0 ± 3.94	227.4 ± 6.09^a^	0.182	0.018	
Change	−18.1 ± 3.71^b^		−3.68 ± 3.70^a^		5.42 ± 4.52^a^				<0.001
HDL-cholesterol (mg/dL)^*∮*^	55.4 ± 2.09	55.8 ± 2.02	58.6 ± 2.55	60.3 ± 2.64	51.1 ± 2.03	53.9 ± 2.60	0.081	0.136	
LDL-cholesterol (mg/dL)^*∮*^	146.5 ± 3.54	129.1 ± 3.50^b,***^	148.0 ± 3.63	142.8 ± 3.55^a^	143.7 ± 3.74	143.3 ± 4.61^a^	0.670	0.017	
Change	−17.4 ± 3.49^a^		−5.14 ± 3.69^a,b^		−0.14 ± 3.77^b^				0.003
Apolipoprotein A-I (mg/dL)^*∮*^	162.8 ± 4.14	161.2 ± 3.50	167.9 ± 5.07	170.5 ± 4.65	152.7 ± 3.59	156.6 ± 4.15	0.065	0.059	
Apolipoprotein B (mg/dL)^*∮*^	126.4 ± 3.85	122.3 ± 3.69	128.7 ± 3.48	127.9 ± 3.29	121.2 ± 3.48	127.6 ± 3.29^*^	0.305	0.349	
Change	−4.06 ± 2.56^b^		−0.85 ± 2.34^a,b^		6.44 ± 2.54^a^				0.010
Glucose (mg/dL)^*∮*^	89.5 ± 1.35	89.3 ± 1.36	88.1 ± 1.11	87.9 ± 1.15	87.6 ± 0.97	86.3 ± 1.23	0.564	0.250	
Insulin (μIU/mL)^*∮*^	8.56 ± 0.51	8.41 ± 0.43	8.95 ± 0.50	8.81 ± 0.52	8.63 ± 0.57	8.70 ± 0.64	0.615	0.895	
hs-CRP (mg/L)^*∮*^	0.53 ± 0.06	0.83 ± 0.16^*^	0.79 ± 0.16	0.75 ± 0.11	0.77 ± 0.13	0.73 ± 0.13	0.253	0.641	
Oxidized LDL (U/L)^*∮*^	63.6 ± 1.98	61.9 ± 1.91	61.6 ± 2.29	61.9 ± 2.66	63.6 ± 2.68	67.9 ± 2.32^**^	0.714	0.084	
Change	−1.74 ± 1.38^b^		0.30 ± 1.55^a,b^		4.27 ± 1.61^a^				0.018

Mean ± SE. ^*†*^Values were estimated from weighed food records and calculated using the database of the computerized Korean food code. ^*‡*^All units of GC-MS data are relative peak area. ^∮^Values were tested by logarithmic transformation. *P* ^*a*^-values derived from ANOVA in baseline. *P* ^*b*^-values derived from ANOVA in follow up. *P* ^*c*^-values derived from ANOVA in changed value. All alphabetical *P* < 0.05 derived from the Bonferroni post hoc test in baseline, follow-up, and changed value, respectively; no significant changes among the each group marked with the same letters and significant changes among the each group marked in different letters. ^*^ *P* <0.05, ^**^ *P* <0.01, ^***^ *P* <0.001 indicate comparisons with baseline values in each group performed with a paired *t*-test

### Plasma fatty acid levels

Relative levels of the following plasma values of PUFAs are shown in Fig. 1 and Table 1: LA (18:2n-6); gamma-linolenic acid (GLA, 18:3n-6); dihomo-gamma-linolenic acid (DGLA, 20:3n-6); arachidonic acid (AA, 20:4n-6); alpha-linolenic acid (ALA, 18:3n-3); eicosapentaenoic acid (EPA, 20:5n-3); and docosahexaenoic acid (DHA, 22:6n-3). Significant effects on overall dietary fat due to changes (difference from baseline) in LA intake between the groups were broadly supported by changes in the plasma LA levels, which were significantly different between groups (*P* < 0.001) (Fig. 1). Plasma LA levels decreased by 12.2% in the low LA group and increased by 13.8% in the high LA group after diet intervention from the baseline values (Fig. 1). The low LA group showed a significant decrease from baseline in plasma LA, GLA and DGLA, whereas the high LA group showed increases in plasma LA, GLA, DGLA and AA, and these changes in plasma LA (*P <* 0.001), GLA (*P =* 0.001), ALA (*P =* 0.018), DGLA (*P <* 0.001) and AA (*P =* 0.015) were significantly different between the groups (Fig. 1 and Table 1). At week 8, plasma LA (Fig. 1), ALA (*P* = 0.004) and DGLA (*P* = 0.008) were higher in the high LA group than that in the low LA group (Table 1).

### Blood pressure, glucose, and serum lipid profiles

The low LA group showed significant decreases from baseline in diastolic blood pressure, total cholesterol and LDL-cholesterol at 8 weeks (Table 1). Changes in total cholesterol (*P* < 0.001) and LDL-cholesterol (*P =* 0.003) were significantly different between groups, and greater reductions in the low LA group were observed than those in the high LA group. The high LA group also showed significant increases in serum apoB and plasma ox-LDL at 8 weeks. The changes in the serum apoB (*P =* 0.010) and plasma ox-LDL (*P =* 0.018) were significantly different between groups, with increases observed in the high LA group and decreases in the low LA group.

### hs-CRP, hemostatic markers and Lp-PLA_2_ activity

The low LA group showed significant increases from baseline in hs-CRP, fibrinogen and aPTT at 8 weeks (Table 1). The high LA group showed significant increases from baseline in plasma Lp-PLA_2_ activity at 8 weeks. The changes in the plasma Lp-PLA_2_ activity were significantly different between groups (*P =* 0.002), with increases observed in the high LA group and decreases in both the medium LA and low LA groups (Fig. 1).

### Correlations among changes in the plasma fatty acid levels, LDL, ox-LDL, apoB, CRP, hemostatic markers and Lp-PLA_2_ activity

**Fig. 2:**
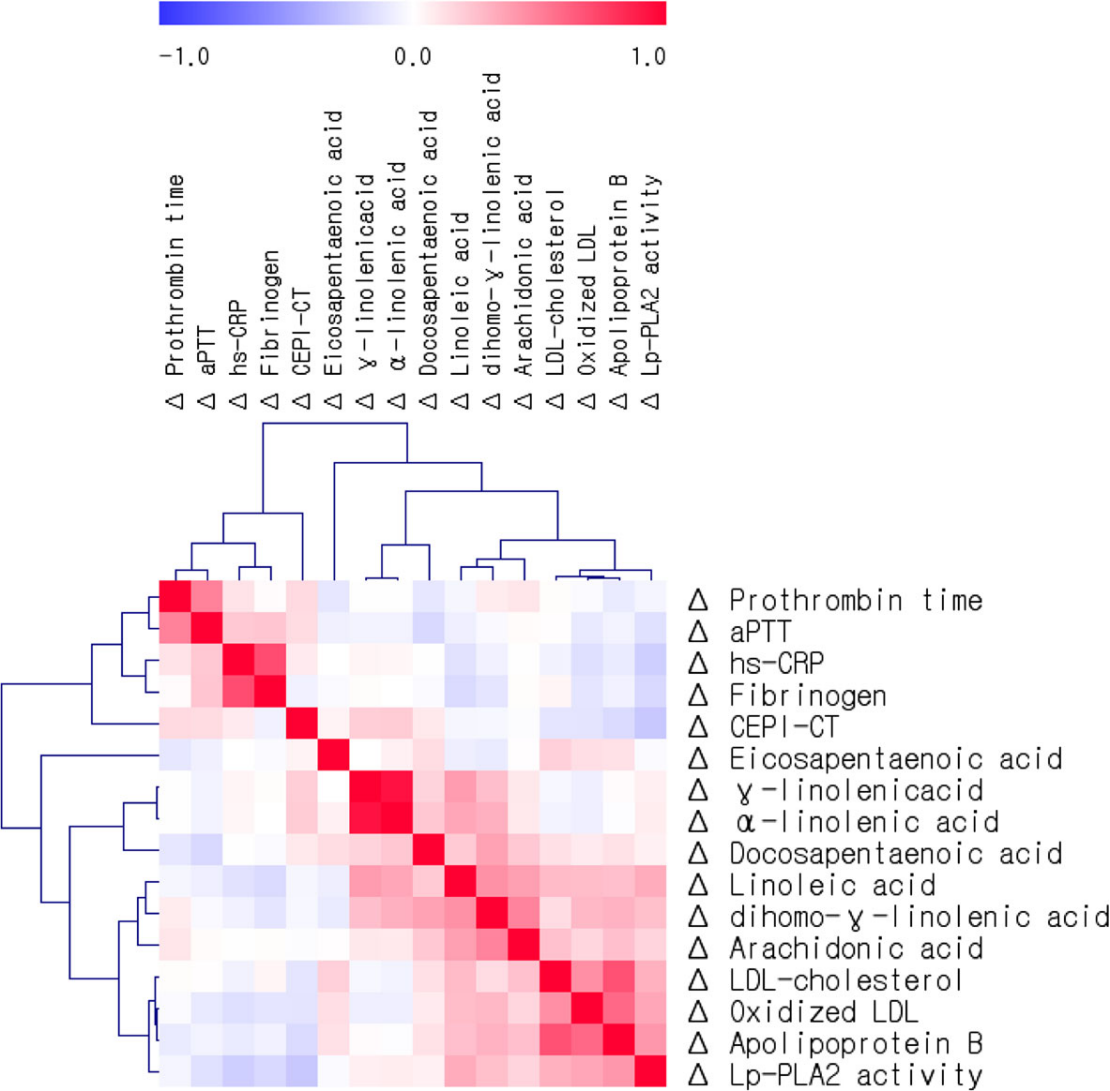
Correlation matrix among changes in plasma fatty acid levels, LDL, ox-LDL, apoB, hs-CRP, hemostatic markers and Lp-PLA_2_ activity. Correlations were obtained using Pearson’s correlation coefficient. *Red* indicates a positive correlation, and *blue* indicates a negative correlation

**Fig. 3:**
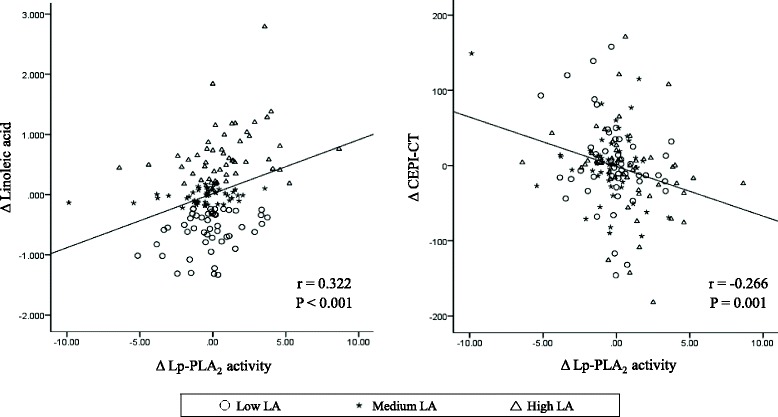
Correlations among changes in Lp-PLA_2_ activity, LA, and CEPI-CT. *r*: Pearson’s correlation coefficients

Figure 2 shows the correlations among changes in plasma fatty acid levels, LDL, ox-LDL, apoB, CRP, hemostatic markers and Lp-PLA_2_ activity in all participants (*n* = 147). The changes in plasma LA were positively correlated with the changes in GLA, ALA, DGLA, AA, Lp-PLA_2_ activity (all *P*-values < 0.001), apoB (*P =* 0.002), ox-LDL (*P =* 0.001) and LDL-cholesterol (*P =* 0.001). The changes in Lp-PLA_2_ activity were positively correlated with the changes in LA (Fig. 3, *r* = 0.322, *P <* 0.001), DGLA (*P =* 0.002), AA (*P =* 0.038), LDL-cholesterol (*P <* 0.001), ox-LDL (*P <* 0.001) and apoB (*P <* 0.001) but were negatively correlated with the changes in CEPI-CT (Fig. 3, *r* = -0.266, *P =* 0.001) and also showed a trend toward a negative correlation with aPTT (*P =* 0.062). Other correlations among plasma fatty acid levels, LDL, ox-LDL, apoB, CRP, hemostatic markers and Lp-PLA_2_ activity are shown in Fig. 2 (*P*-values are available in Additional file 1).

Because the regulation of Lp-PLA_2_ activity is complex, a multiple linear regression analysis was performed to determine the independent effects of the following variables on Lp-PLA_2_ activity: age, BMI, gender, baseline Lp-PLA_2_ and changes in LDL-cholesterol, ox-LDL, LA, apoB, GLA, DGLA, AA, ALA, EPA, and DHA. Lp-PLA_2_ activity was affected by baseline Lp-PLA_2_ activity (β = –0.228; CI: –2.432, –0.511), as well as changes in apoB (β = 0.304; CI: 0.019, 0.058) and plasma LA (β = 0.198; CI: 0.187, 1.358) (R^2^ = 0.255, *P* = 0.010).

## Discussion

This study examined whether changes in LA intake in the form of soy oil over 8 weeks would alter Lp-PLA_2_ activity, which is an independent risk factor for CVD. The results showed that 8 weeks of daily 10 mL supplementation with soy oil, equivalent to approximately 5 mL of LA, increased the plasma LA levels by 14% and Lp-PLA_2_ activity by 6.2% without changes in LDL-cholesterol levels. Thus, the major finding of the present study is that when the total energy intake was kept constant, an increase in plasma LA from intake of soy oil was independently associated with an increase in Lp-PLA_2_ activity in non-diabetic and non-obese healthy subjects who were not taking any medications or supplements that could affect lipid metabolism, platelet function or inflammation.

To date, no dietary intervention studies have examined changes in n-6 fatty acids in the context of Lp-PLA_2_ activity, and only one cross-sectional study with a large population examined the potential association between Lp-PLA_2_ activity and plasma fatty acid levels [12]. Steffen et al. showed that Lp-PLA_2_ mass and activity were significantly higher in participants with greater plasma levels of LA and DGLA [12]. In this study, the change (difference from baseline) in Lp-PLA_2_ activity was associated with changes in plasma n-6 PUFAs, including LA, DGLA and AA; however, a multiple regression analysis revealed that only the change in LA was independently and positively correlated with the change in Lp-PLA_2_ activity—a hallmark of inflammation, atherosclerosis and CVD [2, 12].

As a major unsaturated fatty acid in the diet, LA is considered to be atherogenic because of its pro-oxidative and proinflammatory properties [12, 17, 18]. Thus, the positive association between changes in plasma LA and Lp-PLA_2_ activity in this study may be explained by the influence of LA on inflammation. Hennig et al. [17] also reported that the positive association between LA and Lp-PLA_2_ was due to its promotion of inflammation and the activation of phosphoinositol 3-kinase in cell culture models [17]. Indeed, there is a considerable body of evidence showing that LA affects inflammatory signaling cascades, which may in turn influence Lp-PLA_2_ expression via phosphatidylinositol 3-kinase and p38 mitogen-activated protein kinase. However, further research is needed to determine whether these effects are involved in the induction of Lp-PLA_2_ expression in vivo.

Additionally, an increase in Lp-PLA_2_ activity was also related to increases in apoB and ox-LDL and a decrease in CEPI-CT. Ox-LDL is generated under oxidative stress [19], which is caused by increased LA and Lp-PLA_2_ activity [1, 2, 12, 17, 18]. LA was positively correlated with Lp-PLA_2_ activity in the present study, suggesting that oxidative stress is enhanced in the high LA group. Moreover, Wang et al. [20] demonstrated that ox-LDL stimulated expression of Lp-PLA_2_; therefore, a vicious cycle of ox-LDL generation by elevated LA or Lp-PLA_2_-induced oxidative stress and increased Lp-PLA_2_ due to ox-LDL might occur in the body. CEPI-CT has previously been related to bleeding time [21] and shown to serve as a method to identify high residual platelet reactivity despite aspirin therapy, thereby predicting the risk of ischemic events [22]. Thus, a negative correlation between changes in Lp-PLA_2_ activity and CEPI-CT and a positive correlation between changes in the plasma LA level and Lp-PLA_2_ activity could suggest an increase in the plasma LA level from increased dietary LA intake. Additionally, aPTT, as a functional assay, is accurate and highly reproducible and can identify abnormalities within the coagulation pathway [22]. In this study, the low LA group showed significantly increased aPTT (by 3.5%) at 8 weeks and a trend toward a negative correlation between changes in Lp-PLA_2_ activity and aPTT.

There was an increase in fibrinogen and hs-CRP within the normal range in the low LA group, despite significant reductions in LDL- and total-cholesterol. These findings may indicate a transient phenomenon present during the transition from normal LA intake to low LA intake. Similarly, Tylner et al. [23] observed a negative relationship between changes in fibrinogen and the intake of an energy-dense formula with oleic and LA in older, frail adults with a lower dietary intake than their estimated needs.

The present study has several strengths, including dietary intervention with different levels of LA and the direct measurement of plasma fatty acid levels compared with that of previous studies, as well as indirect measurements of fat intake from dietary recall. Additionally, numerous demographic, lifestyle and clinical factor adjustments were made to better determine whether changes in LA were associated with changes in Lp-PLA_2_ activity. In terms of limitations, the fat intake in the medium LA dietary group (control group) is typical for the Korean population but much lower than that in Western countries. Thus, the results of this study cannot be generalized to other populations with a higher fat intake. Furthermore, it should be noted that the role in Lp-PLA_2_ is still controversial. Despite these limitations, the present findings indicate that higher plasma LA levels, resulting from a higher dietary LA intake, are associated with higher Lp-PLA_2_ activity, as well as increases in apoB and ox-LDL and decreases in CEPI-CT.

## Conclusions

An increase in plasma LA following oral intake of soy oil for 8 weeks elevated apoB, ox-LDL, and Lp-PLA_2_ activity in healthy subjects. Changes in plasma LA positively and independently correlated with changes in Lp-PLA_2_ activity, which was negatively correlated with changes in CEPI-CT. Our results suggest that consumption of n-6 fatty acids, especially LA, is associated with increases in Lp-PLA_2_ activity and CEPI-CT, and these increases may be mediated by elevated oxidative stress.

## Additional file

Additional file 1:
*P*-values for correlations among changes in (Δ) plasma fatty acid level, LDL-cholesterol, oxidized LDL, apolipoprotein B, hs-CRP, hemostatic markers and Lp-PLA_2_ activity. *P*-values were derived from Pearson’s correlation coefficients. (DOCX 29 kb)

## Abbreviations

ALA: Alpha-linolenic acid
ANOVA: One-way analysis of variance
apo: Apolipoprotein
aPTT: Activated partial thromboplastin time
CEPI-CT: Collagen-epinephrine closure time
CVD: Cardiovascular disease
DGLA: Dihomo-gamma-linolenic acid
DHA: Docosahexaenoic acid
EPA: Eicosapentaenoic acid
GC-MS: Gas chromatography mass spectrometry
GLA: Gamma-linolenic acid
HDL: High-density lipoprotein
hs-CRP: High-sensitivity C-reactive protein
LA: Linoleic acid
LDL: Low-density lipoprotein
Lp-PLA2: Lipoprotein-associated phospholipase A2
ox-LDL: Oxidized low-density lipoprotein
PAF-AH: Platelet-activating factor acetylhydrolase
PT: Prothrombin time
