# Supplementary material for: Impact of 8-week linoleic acid intake in soy oil on Lp-PLA2 activity in healthy adults
Source: Nutr Metab (Lond). 2017 May 8;14:32. doi: 10.1186/s12986-017-0186-2 (PMC5422895; doi:10.1186/s12986-017-0186-2)
Supplement: Additional file 1: — P-values for correlations among changes in (Δ) plasma fatty acid level, LDL-cholesterol, oxidized LDL, apolipoprotein B, hs-CRP, hemostatic markers and Lp-PLA2 activity. P-values were derived from Pearson’s correlation coefficients. (DOCX 29 kb) [file 12986_2017_186_MOESM1_ESM.docx]

**Table S1. *P*-values for correlations among changes in (Δ) plasma fatty acid level, LDL-cholesterol, oxidized LDL, apolipoprotein B, hs-CRP, hemostatic markers and Lp-PLA_2_ activity.**

|  |  | *P*-values | | | | | | | | | | | | | | | |
| --- | --- | --- | --- | --- | --- | --- | --- | --- | --- | --- | --- | --- | --- | --- | --- | --- | --- |
|  |  | 1 | 2 | 3 | 4 | 5 | 6 | 7 | 8 | 9 | 10 | 11 | 12 | 13 | 14 | 15 | 16 |
| 1 | Δ Prothrombin time | - |  |  |  |  |  |  |  |  |  |  |  |  |  |  |  |
| 2 | Δ Activated partial thromboplastin time | **<0.001** | - |  |  |  |  |  |  |  |  |  |  |  |  |  |  |
| 3 | Δ hs-CRP | 0.186 | **0.008** | - |  |  |  |  |  |  |  |  |  |  |  |  |  |
| 4 | Δ Fibrinogen | 0.810 | **0.006** | **<0.001** | - |  |  |  |  |  |  |  |  |  |  |  |  |
| 5 | Δ CEPI-CT | 0.072 | 0.086 | 0.333 | 0.426 | - |  |  |  |  |  |  |  |  |  |  |  |
| 6 | Δ Eicosapentaenoic acid (C20:5, n-3) | 0.182 | 0.397 | 0.993 | 0.795 | 0.548 | - |  |  |  |  |  |  |  |  |  |  |
| 7 | Δ γ-linolenic acid (C18:3, n-6) | 0.930 | 0.489 | 0.625 | 0.876 | **0.018** | 0.989 | - |  |  |  |  |  |  |  |  |  |
| 8 | Δ α-linolenic acid (C18:3, n-3) | 0.945 | 0.486 | 0.643 | 1.000 | **0.014** | 0.510 | **<0.001** | - |  |  |  |  |  |  |  |  |
| 9 | Δ Docosahexaenoic acid (C22:6, n-3) | 0.503 | 0.935 | 0.704 | 0.586 | 0.993 | **<0.001** | 0.555 | 0.335 | - |  |  |  |  |  |  |  |
| 10 | Δ Linoleic acid (C18:2, n-6) | 0.631 | 0.353 | 0.095 | **0.037** | 0.611 | 0.331 | **<0.001** | **<0.001** | 0.240 | - |  |  |  |  |  |  |
| 11 | Δ Dihomo-γ-linolenic acid (C20:3, n-6) | 0.368 | 0.769 | 0.449 | 0.137 | 0.658 | 0.244 | **0.002** | **<0.001** | **0.043** | **<0.001** | - |  |  |  |  |  |
| 12 | Δ Arachidonic acid (C20:4, n-6) | 0.233 | 0.832 | 0.960 | 0.877 | 0.909 | 0.895 | 0.256 | 0.284 | **<0.001** | **<0.001** | **<0.001** | - |  |  |  |  |
| 13 | Δ LDL-cholesterol | 0.888 | 0.922 | 0.483 | 0.621 | 0.141 | **0.025** | 0.678 | 0.539 | **0.007** | **0.001** | 0.092 | **0.003** | - |  |  |  |
| 14 | Δ Oxidized LDL | 0.859 | 0.213 | 0.067 | 0.131 | 0.128 | 0.126 | 0.391 | 0.366 | 0.062 | **0.001** | **0.001** | 0.052 | **<0.001** | - |  |  |
| 15 | Δ Apolipoprotein B | 0.273 | 0.512 | 0.253 | 0.428 | **0.034** | 0.150 | 0.790 | 0.950 | **0.006** | **0.002** | **<0.001** | **0.002** | **<0.001** | **<0.001** | - |  |
| 16 | Δ Lp-PLA_2_ activity | 0.569 | 0.077 | **0.005** | **0.028** | **0.001** | 0.836 | 0.439 | 0.392 | 0.440 | **<0.001** | **0.002** | **0.038** | **<0.001** | **<0.001** | **<0.001** | - |

*P*-values were derived from Pearson’s correlation coefficients.
